# The plastid genome of a narrowly distributed species *Artocarpus petelotii* (Moraceae)

**DOI:** 10.1080/23802359.2020.1871434

**Published:** 2021-02-11

**Authors:** Huanhuan Chen, Qing Liu

**Affiliations:** a College of Biological Resource and Food Engineering, Qujing Normal University, Qujing, China; b Key Laboratory of Yunnan Province Universities of the Diversity and Ecological Adaptive Evolution for Animals and Plants on YunGui Plateau, Qujing Normal University, Qujing, China; c School of Resources and Environment, Baoshan University, Yunnan, China

**Keywords:** *Artocarpus petelotii*, chloroplast genome, phylogenetic analysis

## Abstract

The complete chloroplast genome sequence of *Artocarpus petelotii* was determined, a narrowly distributed species at high altitudes in the family Moraceae. To better determine its phylogenetic location with respect to the other Moraceae species, the complete plastid genome of *A. petelotii* was sequenced. The whole chloroplast genome is 161,009 bp in length, consisting of a pair of inverted repeat (IR) regions of 25,682 bp, one large single-copy (LSC) region of 89,552 bp, and one small single-copy (SSC) region of 20,093 bp. The overall GC content of the whole chloroplast genome is 35.8%. Further, maximum likelihood phylogenetic analysis was conducted using 26 complete plastomes of the Moraceae, which support close relationships among *A. petelotii*, *A. nanchuanensis* and *A. heterophyllus*.

*Artocarpus petelotii* Gagnep is a member of the genus *Artocarpus* (Moraceae), which includes approximately 70 species mostly native to the hot regions of South and Southeast Asia and Oceania (William et al. 2017). *Artocarpus petelotii* is a narrowly distributed species at high altitudes in Southeast Yunnan of China and North Vietnam (Wu and Zhang 1989). The fruits of *A. petelotii* contains plenty of nutritional compositions with potential great medicinal value (Ren et al. 2014). For a better understanding of the relationships of *A. petelotii* and other Moraceae species, it is necessary to reconstruct a phylogenetic tree based on high-throughput sequencing approaches.

Fresh young leaves of *A. petelotii* in Xishuangbanna (Yunnan, China; Long. 101.2611 E, Lat. 21.9275 N, 555 m) were picked for DNA extraction (Doyle and Dickson 1987). The voucher was deposited at the Biodiversity Research Group of Xishuangbanna Tropical Botanical Garden (Accession Number: XTBG-BRG-10003). Long-range polymerase chain reaction was carried out following Zhang et al. (2016), with their 15 pairs of universal primers. The 15 purified polymerase chain reaction products were mixed with 0.4 μg for each. A total of 6 μg product was sent to BGI, Shenzhen for library construction and genome sequencing on the Illumina Hiseq 2000 Platform (Illumina, San Diego, CA), more than 100 Mb of sequence data for the sample was obtained and subjected to chloroplast gnome assmembly. Paired-end reads were assembled using the trial version of CLC v.8 (http://www.qiagenbioinformatics.Com). The contigs were aligned using the publicly available plastid genome of *Morus celtidifolia* (Accession number MT154045). The complete chloroplast genome was annotated in Geneious 10.1.3 (Kearse et al., 2012).

The chloroplast genomes of *A. petelotii* (Accession number MW250918), with a length of 161,009 bp, was 257 bp, 622 bp, 1524 bp and 2550 bp larger than that of *A. nanchuanensis* (160,752 bp, LAU10104), *A. heterophyllus* (160,387 bp, MG434693), *Morus celtidifolia* (159,485 bp, MT154045) and *M. mongolica* (158,459 bp, KM491711). It was also 1161 bp smaller than that of *Broussonetia kurzii* (162,170 bp, MH118529). The length of the inverted repeats (IRs), large single-copy (LSC), and small single-copy (SSC) regions of *A. petelotii* was 25,682 bp, 89,552 bp, and 20,093 bp, respectively. The overall G + C content is 35.8% (LSC, 33.4%; SSC, 28.6%; IR, 42.8%).

Furthermore, based on 16 previously reported plastomes from Genbank including *A. heterophyllus*, *M. mongolica*, *M. alba* var. *Atropurpurea*, *M. alba* var. *multicaulis*, *M. notabilis*, *M. indica*, *M. celtidifolia*, *B. kazinoki*, *B. monoica*, *B. kaempferi*, *B. papyrifera*, *B. kurzii*, *Ficus carica*, *Ficus hirta*, *Ficus religiosa*, *Ficus racemosa*, 3 plastome sequences reported in our previous study including *A. nanchuanensis*, *Ficus tinctoria*, *Ficus altissima* and 6 published plastome genome sequences (Bruun-Lund et al. 2017) including *Mesogyne insignis*, *Castilla elastica*, *Helicostylis tomentosa*, *Naudeopsis krukowii*, *Antaris toxicaria*, *Poulsenia armata*, we reconstructed a phylogenetic tree (Figure 1) with *Ficus tinctoria*, *Ficus carica*, *Ficus hirta*, *Ficus religiosa*, *Ficus altissima*, *Ficus racemosa* as outgroups. Maximum likelihood (ML) phylogenetic analysis were performed base on TVM + F+R2 model in the iqtree version 1.6.7 program with 1000 bootstrap replicates (Nguyen et al. 2015). The ML phylogenetic tree with 58–100% bootstrap values at each node supported that *A. petelotii* was closely related to *A. nanchuanensis* and *A. heterophyllus*.

**Figure 1.**
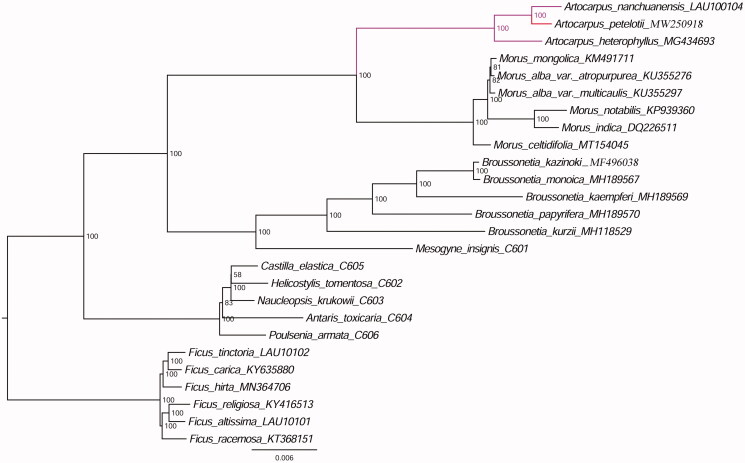
The ML phylogenetic tree for *Artocarpus petelotii* based on other 25 species plastid genomes.

## Data Availability

The data that support the finding of this study are openly available in GenBank of NCBI at https://www.ncbi.nlm.nih.gov, reference number MW250918.
